# Complications of Veress Needle Versus Open Technique in Abdominal Surgeries

**DOI:** 10.7759/cureus.14926

**Published:** 2021-05-09

**Authors:** Ghassan I Alhajress, Ibrahim Al Babtain, Abdullah Alsaghyir, Hassan Arishi

**Affiliations:** 1 College of Medicine, King Saud Bin Abdulaziz University for Health Sciences, Riyadh, SAU; 2 College of Medicine, King Abdullah International Medical Research Center, Riyadh, SAU; 3 Department of Surgery, Ministry of National Guard - Health Affairs, Riyadh, SAU

**Keywords:** abdominal surgery, veress needle, pneumoperitoneum

## Abstract

Introduction

In any laparoscopic procedure, the first and most important step is abdominal entry. This is followed by the creation of pneumoperitoneum, which is essential for lifting the abdominal wall off of the internal organs and visualizing the entered space. However, the entry and establishment of pneumoperitoneum are not without risks and complications, the most serious of which include vascular injuries and bowel perforations in all the different techniques used. The most commonly used techniques for abdominal entry are the closed (Veress) and open (Hasson) techniques, the choice of which varies according to the surgeon’s preference and other regional and local factors.

Aim

To compare the outcomes between the open technique and the Veress needle for accessing the peritoneum during laparoscopic surgery.

Methodology

This was a retrospective cohort study that aimed to compare the outcomes between the Veress needle and the open technique for entering the peritoneum in laparoscopic surgeries. A chart review was used as an instrument to collect data. The study was conducted in King Abdul-Aziz Medical City, Riyadh, Saudi Arabia. All patients who underwent abdominal laparoscopic surgery from 2006 to 2016 were included.

Results

We analyzed 365 patients who underwent laparoscopic abdominal surgery. The mean age of the patients was 32.6 years. The most common postoperative complication occurring during the admission period was abdominal pain (40.5%). Postoperative complications during admission were significantly associated with the type of needle used (χ2=10.641; p=0.001).

Conclusion

The type of technique used for entry and peritoneal access was associated with the occurrence of postoperative complications in abdominal surgeries. Thus, the choice of open or Veress technique for peritoneal access should be individualized based on factors such as patient sex, clinical diagnosis, and most importantly, the surgeons’ experience and preference.

## Introduction

Laparoscopy was first performed in humans in 1910 by Jacobeus in Sweden [1]. In the last few decades, laparoscopy has evolved considerably and is now a commonly preferred procedure in many surgical specialties because of its advantages over traditional laparotomy [2]. Although laparoscopy has many advantages over laparotomy, it is not devoid of complications, most of which are associated with the entry into the surgical site [2,3-5]. In any laparoscopic procedure, the first and most important step is entry. Entry is followed by establishing a working space, usually through the creation of pneumoperitoneum, which is essential to lift the abdominal wall off of the internal organs and visualize the entered space, thus providing surgeons greater freedom to manipulate laparoscopic instruments under more direct vision [3]. However, entering and establishing pneumoperitoneum is not without risks and complications, the most serious of which include vascular injuries (0-0.2%) and bowel perforations (0.1-0.2%) in all the techniques used [3,6]. A major impediment toward patient recovery is the late recognition of such injuries, especially bowel perforations (30-50%), as they are usually discovered post-op when they present with peritonitis and intra-abdominal abscesses, which significantly increase the morbidity and mortality of patients (0.003%) [3,6]. Other complications include urologic injury, hepatic injury, and carbon dioxide embolism (0.001%) [2]. Many different techniques, methods, and instruments have been described in the literature in an attempt to reduce or eliminate these complications; however, none has been proven to be universally effective to date [2,7].

Many techniques and aids have been used and studied with regard to the entry step in laparoscopic procedures. These include open (Hasson), closed (Veress), direct trocar insertion, disposable shielded trocars, radially expanding trocars, and visual entry systems [3]. Among these, the most used techniques are closed (Veress) and open (Hasson), the choice of which varies according to the surgeon’s preference and other regional and local factors [2]. Interestingly, Janos Veress from Hungary developed the Veress needle in 1938 for the induction of pneumothorax, and not for laparoscopic usage [8]. Veress use was first popularized in France in 1947 [9] and is now the most widely used technique, especially for gynecological surgeries [1,2]. In 1971, Hasson described the open technique for the first time as a way of avoiding a few of the complications associated with the Veress technique [10].

The complication rates vary according to the entry method and device used, and accordingly, their reported incidence varies widely in the literature [3]. Some studies, such as this example by Bonjer et al., report a higher rate of complications with closed laparoscopy: “the rates of visceral and vascular injury were respectively 0.08% and 0.07% after closed laparoscopy, and 0.05% and 0% after open laparoscopy” [11]. On the other hand, the Swiss Association for Laparoscopic and Thoracoscopic Surgery stated in their study that “the open-access method used in the current series failed to show any superiority over the closed establishment of pneumoperitoneum.” Other studies such as those by Molloy et al. identified the surgical specialty as the only factor affecting the rates of complications associated with each method, with bowel complications occurring at a rate of 0.4/1000 with gynecologists and 1.5/1000 with general surgeons [12]. In the Society of Obstetricians and Gynaecologists of Canada Clinical Practice Gynecology Guidelines, the final conclusion was that “there is no evidence that the open entry technique is superior to, or inferior to the other entry techniques currently available.” Therefore, the debate continues as to which technique is superior to the other, and what local practice and patient factors are involved [2,3,13].

As the debate goes on, and the preference and experience of the surgeon continue to be the decisive factor in choosing one method over the other, it is crucial to look into the local patient population of the Kingdom of Saudi Arabia, and the outcome of each method in relation to the complication rates and extent of use of each technique. Most studies have explored immediate and short-term complications, and rarely examined long-term complications such as hernia. This study aims to investigate the risk of hernia, as well as the important immediate complications, such as bowel injury and vascular injury, in relation to patient factors and techniques. As a result, we hope to aid surgeons in the choice of which entry method to adopt, that is associated with the least incidence of local complications.

## Materials and methods

This was a retrospective cohort study that aimed to compare the outcomes between the Veress needle and open technique for entering the peritoneum in laparoscopic surgeries. A chart review was used as an instrument to collect data, which was the ideal method to achieve the study goals. The study design allowed us to evaluate all possible complications of both techniques in a comparatively large sample size with little to no cost. It also helped in understanding the best and safest laparoscopic surgery technique. The study was conducted in King Abdul-Aziz Medical City, Riyadh, Saudi Arabia. All patients who underwent abdominal laparoscopic surgery from 2006 to 2016 were included. The exclusion criteria included patients with a history of more than one abdominal surgery, history of hernia, age <20 years or >50 years, and those who underwent emergency surgeries. The variables included in the study: early surgical complications, late surgical complications, type of surgery, age, gender, and comorbidities.

## Results

**Figure 1:**
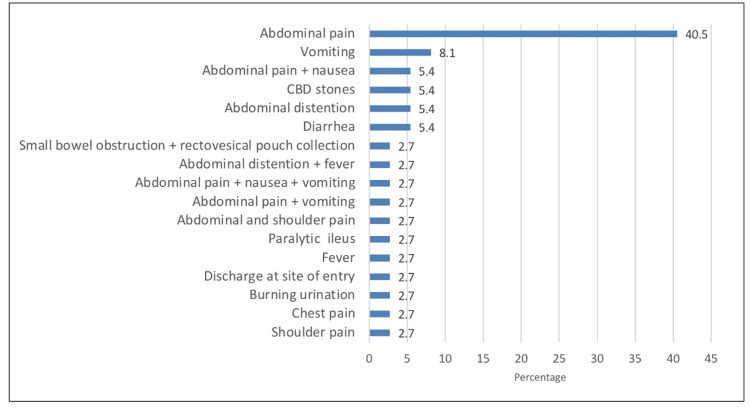
Postoperative complications during admission CBD, common bile duct

**Figure 2:**
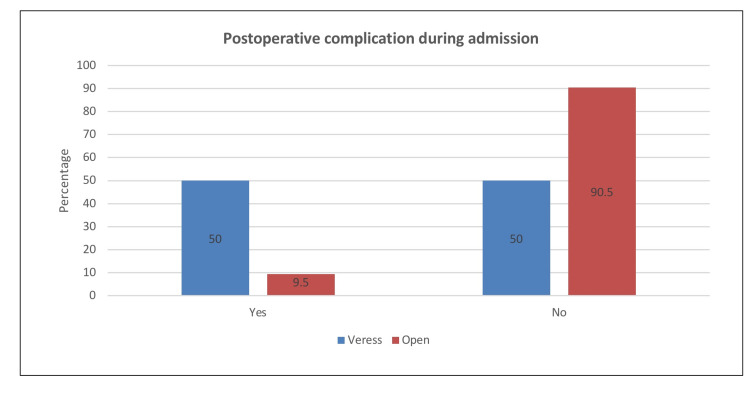
Type of needle used in patients with postoperative complications during admission

**Figure 3:**
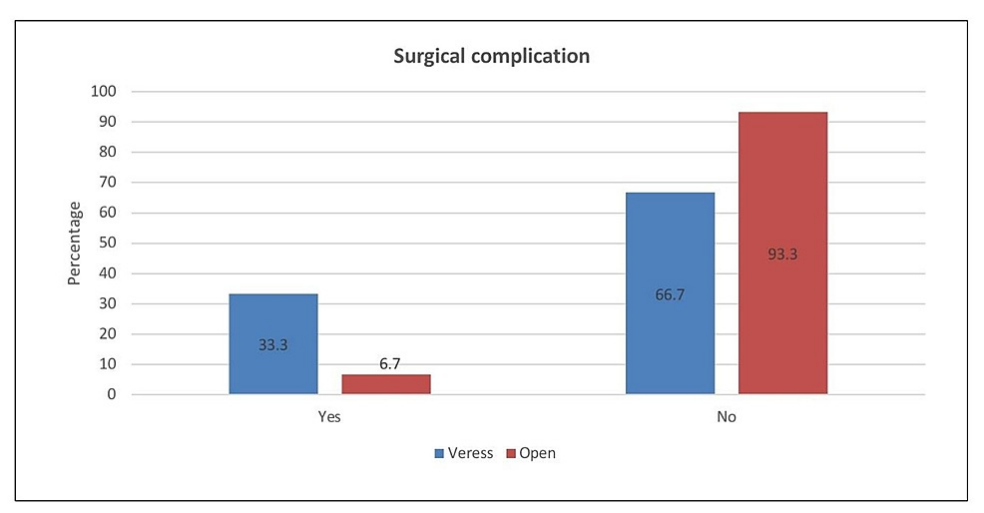
The relationship between surgical complications and the type of needle used

We analyzed 365 patients who underwent laparoscopic abdominal surgery. As shown in Table 1, the mean age of the patients was 32.6 years, while the mean values of weight (kg), height (cm), and body mass index (BMI) (kg/m2) were 76.7, 162.3, and 28.8, respectively. Furthermore, there were slightly more females (51.8%) than males (48.2%). The mean values of gravida, para, and abortion were 4.32, 4.02, and 1.97, respectively. With respect to patient comorbidities, the proportion of patients with hypertension, diabetes, vascular comorbidities, previous hernia, and immunocompromised state were 5%, 5%, 3.8%, 0.8%, and 0.3%. Of those with vascular comorbidities, nine (75%) had dyslipidemia, one was diagnosed with ventricular septal defect, and the other two had varicose veins and paroxysmal atrial fibrillation. Of those patients with a previous hernia, three were umbilical and one was inguinal. The other commonly reported comorbidities included asthma (5.5%), followed by hypothyroidism (3.6%). As for immediate postoperative complications, the prevalence of patients with bowel injury was 2.5%. Three cases occurred during bowel entry. We also observed two cases of vascular injury with one case during entry, which were reported as bleeding. In addition, there were three cases of omental injury with two cases during entry. Moreover, one case was reported to have gas leakage, and two cases had surgical site infection during admission. The most commonly reported postoperative complications that occurred within the admission period (Figure 1) were abdominal pain (40.5%) and vomiting (8.1%). Our investigation revealed that the proportion of patients who visited the emergency room for surgical site complications was 10.1%; the most commonly reported complication was pain (51.4%) and a combination of pain and discharge (13.5%). Two patients complained of surgical site hernia, and 7.1% presented with surgical complications. The most common surgical complication was abdominal pain (23.8%). Additionally, the mean number of outpatient visits was 2.01 (Figure 2). Postoperative complication occurring within the admission period was found to have a significant relationship with the type of needle used (χ2=10.641; p=0.001). As shown in Figure 3, surgical complications showed a significant relationship with the type of needle used (χ2=6.334; p=0.012).

**Table 1:** Basic demographic profile of the patients (n=365)

Study variables	Mean ± SD
Age in years	32.6 ± 9.26
Gravida (n=34)	4.32 ± 2.25
Para (n=60)	4.02 ± 2.57
Abortion (n=32)	1.97 ± 1.33
Weight (kg)	76.7 ± 18.6
Height (cm)	162.3 ± 13.3
BMI (kg/m^2^)	28.8 ± 6.95
Sex	N (%)
Male	176 (48.2%)
Female	189 (51.8%)

## Discussion

Multiple techniques of entry have been described in the literature, but there is still debate as to whether the open or closed technique is superior in terms of risk of complications. In our study, we aimed to compare the outcomes of both techniques used for accessing the peritoneum, in terms of their short- and long-term complications. However, further multicenter studies with larger sample sizes are needed to determine the safety and applicability of each technique.

In our study, there was no bowel injury seen with the closed technique. However, postoperative complications during the admission period were significantly higher with the closed technique (χ2=10.641; p=0.001). When we compared both techniques at six months' postoperatively, we found that the Veress needle technique was associated with more surgical complications (χ2=6.334; p=0.012). This was a finding similar to that of a study by Taye et al., where major complications, including failure to establish pneumoperitoneum, emphysema, perforation, mesenteric vascular injury, and death, were relatively higher with the closed technique. Their study compared 1500 cases of the closed technique with 1500 cases of the open technique. When both techniques were compared in terms of minor complications, there were no significant differences.

In our study, there were nine cases of bowel injury, three of which occurred during entry. There was one case of bleeding during entry, three cases of omental injury, and two cases of surgical site infection during admission [13]. Jansen et al., in their clinical trial comparing both techniques in terms of complications, found that the rate of complications associated with the open technique was 0.17%, while that with the closed technique was 0.07%. Furthermore, they found that complications associated with peritoneal access were significantly higher with the open technique [5]. Compeau et al. aimed to identify preferences for techniques of laparoscopic entry among Canadian general surgeons in their study. Out of 1000, 248 registered surgeons of the Canadian Association of General Surgeons responded [14]. It has been reported that more than 50% of laparoscopic complications are related to peritoneal access [14]. A study by Pickersgill et al. reported that out of 647 cases, less than 5% had gas leakage [15]. In our study, meanwhile, we had only one instance of gas leakage. Schäfer et al. evaluated 26 major vascular injuries, of which only four (15%) cases were performed by inexperienced surgeons (surgeons who had performed less than 50 laparoscopic surgeries) and the rest were performed by experienced or very experienced surgeons (surgeons who had performed more than 50 laparoscopic surgeries) [16].

## Conclusions

The findings of our study suggest that the open technique is relatively safer and associated with fewer complications. However, due to the limited sample size, further research should be conducted. No single technique is considered suitable for all cases. In conclusion, the choice of technique for peritoneal access should be individualized, based on factors such as such as patient sex, diagnosis, and most importantly, the surgeons’ experience and preference.
